# Highly Tunable Cascaded Metasurfaces for Continuous Two‐Dimensional Beam Steering

**DOI:** 10.1002/advs.202300542

**Published:** 2023-06-20

**Authors:** Lingyun Zhang, Li Zhang, Rongbo Xie, Yibo Ni, Xiaoyu Wu, Yuanmu Yang, Fei Xing, Xiaoguang Zhao, Zheng You

**Affiliations:** ^1^ Department of Precision Instrument Tsinghua University Beijing 100084 China; ^2^ State Key Laboratory of Precision Measurement Technology and Instruments Tsinghua University Beijing 100084 China

**Keywords:** beam steering, cascaded metasurfaces, tunable metasurfaces

## Abstract

Cascaded metasurfaces can exhibit powerful dynamic light manipulation by mechanically tuning the far‐field interactions in the layers. However, in most current designs, the metasurfaces are separated by gaps smaller than a wavelength to form a total phase profile, representing the direct accumulation of the phase profiles of each layer. Such small gap sizes may not only conflict with the far‐field conditions but also pose great difficulties for practical implementations. To overcome this limitation, a design paradigm taking advantage of a ray‐tracing scheme that allows the cascaded metasurfaces to operate optimally at easily achievable gap sizes is proposed. Enabled by the relative lateral translation of two cascaded metasurfaces, a continuous two‐dimensional (2D) beam‐steering device for 1064 nm light is designed as a proof of concept. Simulation results demonstrate tuning ranges of ±45° for biaxial deflection angles within ±3.5 mm biaxial translations, while keeping the divergence of deflected light less than 0.007°. The experimental results agree well with theoretical predictions, and a uniform optical efficiency is observed. The  generializeddesign paradigm can pave a way towards myriad tunable cascaded metasurface devices for various applications, including but not limited to light detection and ranging (LiDAR) and free space optical communication.

## Graphical abstract

Far‐field interactions in tunable cascaded metasurfaces are investigated to perform continuous two‐dimensional beam steering with a widetuning range and near‐zero divergence. The proposed design paradigm may provide more possibilities for manipulating electromagnetic waves from visible light to millimeter waves, and may inspire more intriguing applications for other artificially engineered materials.

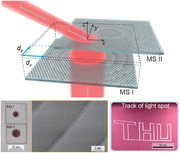

## 1 Introduction

Dynamic light beam manipulation is crucial for many optical applications, including free‐space optical communication,^[^ ^1^ ^]^ light detection and ranging (LiDAR),^[^ ^2^ ^]^ holography,^[^ ^3^ ^]^ etc. While conventional light beam steering based on mechanical rotation and microelectromechanical mirrors^[^ ^4^, ^5^ ^]^ has proven to be useful in many important applications, nanophotonics provides a promising pathway to improve the ability of manipulating optical beams through the development of optical phase arrays,^[^ ^6^ ^]^ focal plane switch arrays,^[^ ^7^ ^]^ and artificially engineered materials, for example, metamaterials and metasurfaces.^[^ ^8^, ^9^ ^]^ As a two‐dimensional (2D) branch of metamaterials, metasurfaces consist of arrays of planarly arranged subwavelength unit cells with spatially varying geometries.^[^ ^10^ ^]^ Through local light–matter interactions, each unit cell demonstrates independent control of amplitude, phase, and polarization for the incident wave, leading to unprecedented beam forming functionalities such as diffraction‐limited focusing,^[^ ^11^ ^]^ achromatic imaging,^[^ ^12^, ^13^ ^]^ optical vortex generation,^[^ ^14^ ^]^ and high‐efficiency holography.^[^ ^15^ ^]^ To meet the growing demand for dynamic beam manipulation,^[^ ^3^ ^]^ tunable (or active) metasurfaces have been investigated using various methods and materials, including electrical gating,^[^ ^16^, ^17^ ^]^ phase‐changing materials,^[^ ^18^ ^]^ micro‐/nanoelectromechanical systems (MEMS/NEMS),^[^ ^19^ ^]^ stretchable substrate,^[^ ^20^ ^]^ and cascaded metasurfaces.^[^ ^21^, ^22^, ^23^, ^24^, ^25^, ^26^, ^27^, ^28^, ^29^, ^30^, ^31^, ^32^, ^33^, ^34^ ^]^ Among these, the cascaded metasurfaces tune the light beam by mechanical motion (translation^[^ ^21^, ^22^, ^23^, ^24^, ^25^, ^26^, ^27^ ^]^ and rotation^[^ ^28^, ^29^, ^30^, ^31^, ^32^, ^33^, ^34^ ^]^) of the cascaded layers and provide a highly promising design framework with advantages of large tunability, easy fabrication, and low cost compared with other tuning mechanisms.^[^ ^35^ ^]^

The tunability of cascaded metasurfaces originates from dynamically tailoring the near‐ or far‐field interactions in the layers. In the near‐field coupling configuration,^[^ ^28^, ^29^, ^30^ ^]^ layers of metasurfaces are stacked closely with gap sizes smaller than the operating wavelength to form the electric and magnetic coupling^[^ ^28^, ^29^, ^36^, ^37^, ^38^ ^]^ and evanescent field coupling.^[^ ^30^, ^39^ ^]^ The resonant nature of metasurfaces makes it possible that even fine‐tuning of the topology and geometry of the resonant structure can give rise to great changes in the device's light modulation function. In the far‐field coupling configuration,^[^ ^21^, ^22^, ^23^, ^24^, ^25^, ^26^, ^27^, ^31^, ^32^, ^33^, ^34^ ^]^ each individual layer of the cascaded metasurfaces modulates the propagation of light separately, and the overall modulation function is a simple combination of individual layer functions. Thus, any mechanical motion that alters the overall modulation function will cause changes in the final output light beam. Based on this configuration, varifocal Moiré lenses^[^ ^31^, ^32^, ^33^ ^]^ and Alvarez lenses,^[^ ^22^, ^23^, ^24^, ^25^ ^]^ 1D tunable beam steering,^[^ ^26^, ^27^, ^34^ ^]^ etc., have been previously demonstrated.

However, for cascaded metasurfaces tuned by lateral motion and based on the far‐field coupling scheme,^[^ ^22^, ^23^, ^24^, ^25^, ^26^, ^27^, ^31^, ^32^, ^33^, ^34^ ^]^ one major assumption often made in research is that the phase profiles of each layer are added directly to perform the overall desired function,^[^ ^22^, ^23^, ^24^, ^25^, ^26^, ^27^, ^33^, ^34^ ^]^ while this phase accumulation assumption is only accurate when the gap between the layers is infinitely small or equal to the Talbot length,^[^ ^40^ ^]^ which is defined as *L*_Talbot_ = 2*P* ^2^/*λ*_0_, where *P* is the lattice constant of metasurfaces and *λ*_0_ is the free‐space wavelength. *L*_Talbot_ is smaller than *λ*_0_ as long as *P* < $\sqrt{2}$ *λ*_0_/2 ≈ 0.7*λ*_0_. Such a small gap size may not only lead to unwanted near‐field coupling but also pose great difficulties in layer alignment and motion control. As a result, many tunable cascaded metasurface devices operate under suboptimal conditions with oversized gaps,^[^ ^22^, ^23^, ^24^, ^25^, ^33^ ^]^ or stay in the numerical simulation stage.^[^ ^26^, ^27^ ^]^ Although efforts have been made to realize rotational Moiré lenses with a gap size of several wavelengths,^[^ ^31^, ^32^ ^]^ a versatile design paradigm entailing arbitrarily large gap sizes is highly desired and remains an outstanding open issue.^[^ ^35^ ^]^

Researchers control the gap sizes in static cascaded metasurfaces through delicate microfabrication processes, as previously reported.^[^ ^41^, ^42^, ^43^, ^44^, ^45^, ^46^ ^]^ In this way, cascaded metasurface devices with fixed relative positions and gap sizes close or smaller than a wavelength are developed to realize multiwavelength achromatic optical elements,^[^ ^41^, ^42^, ^43^ ^]^ where the criterion of direct phase accumulation is met. Devices with gap sizes much larger than a wavelength are used to minimize monochromatic aberrations^[^ ^44^, ^45^ ^]^ and implement a retroreflector,^[^ ^46^ ^]^ where the reiterative ray‐tracing method is proven to be effective for phase profile optimization.

Inspired by the design methods of static cascaded metasurfaces with large gap sizes, we propose a design paradigm for tunable cascaded metasurfaces with arbitrarily prespecified gap sizes. The paradigm incorporates a numerical unit cell model and a theoretical ray‐tracing scheme based on the generalized Snell's law of refraction in full space. In the ray‐tracing scheme, a reverse ray‐tracing technique is utilized to design the phase profiles of the cascaded metasurfaces, and a forward ray‐tracing simulation is performed to evaluate the performance of the designed device. As a practical example, a tunable cascaded metasurface device for continuous 2D beam steering at the near‐infrared wavelength of 1064 nm is designed and simulated. By converting relative lateral translation to the rotation of the light propagation direction, the designed device demonstrates tuning ranges of ±45° for biaxial deflection angles within biaxial translations of ±3.5 mm, while the deflected beam remains near‐zero divergence at the prespecified gap size. Experimental results show that the beam‐steering performance of fabricated devices agrees well with the theoretical predictions, and exhibits good uniformity in the output optical power efficiency during the tuning process. This work thereby provides a universal platform to design and optimize dynamic cascaded metasurfaces with arbitrary overall phase profiles for various applications, including but not limited to beam steering, lensing, and beam shaping.

## 2 Results

### 2.1 Principle and Design of the Cascaded Metasurfaces for Beam Steering

**Figure 1:**
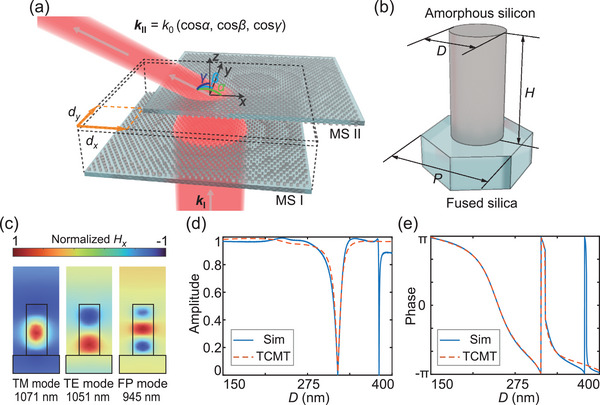
Design of the cascaded metasurfaces and unit cell structure. a) Schematic diagram of two cascaded metasurfaces for beam steering, consisting of an input metasurface (MS I) and output metasurface (MS II), with relative lateral translations *d_x_* and *d_y_*. The cascaded metasurfaces steer the incident beam with wave vector ***k***_**I**_ such that it is transmitted as a beam with wave vector ***k***_**I** **I**_. b) Schematic of one hexagonal metasurface unit cell with lattice constant *P* containing an amorphous silicon cylinder with diameter *D* and height *H* patterned on a fused silica substrate. c) Simulated normalized magnetic field distributions along *x*‐axis (*H_x_*) of the metasurface unit cell with a lattice constant *P* = 560 nm, diameter *D* of 250 nm, and height *H* of 600 nm. The transverse magnetic (TM), transverse electric (TE), and Fabry‒Perot (FP) resonance modes occur at the free‐space wavelength of 1071, 1051, and 945 nm, respectively. d,e) Amplitude and phase of the transmission coefficient of the unit cell versus *D* under normal incidence, with *P* and *H* fixed to 560 and 600 nm, respectively. The results are calculated by numerical simulation (Sim) and temporal coupled mode theory (TCMT).

Our design paradigm incorporates the principle of continuous 2D beam steering enabled by two cascaded metasurfaces placed parallel to the *x–y* plane, as illustrated in **Figure** **1**a. The optical beam successively passes through the input metasurface (MS I) and output metasurface (MS II). The relative lateral translations of MS II with respect to MS I, *d_x_*, and *d_y_*, allow biaxial rotation of the propagation direction of the outgoing beam with respect to the incident beam. Specifically, without loss of generality, the incident and outgoing wave vectors are expressed as ***k*_I_** and ***k*_II_** = *k*_0_ (cos *α*, cos *β*, cos *γ*), where *k*_0_ = 2*π*/*λ*_0_, *λ*_0_ is the vacuum wavelength of the optical beam and *α*, *β*, and *γ* are the direction angles with respect to the *x‐*, *y‐*, and *z‐*axes, respectively. Only two of the three direction angles are independent since cos^2^ *α* + cos^2^ *β* + cos^2^ *γ* = 1. According to this definition, when *α = β* = 90°, the output optical beam travels along the *z*‐axis, i.e., *γ =* 0. Besides, the deflection angles can be calculated as *α* – 90° and *β* – 90°. For the purpose of continuous beam steering, the ideal phase profiles of MS I and MS II should allow the three direction angles *α*, *β*, and *γ* to be continuous functions of *d_x_* and *d_y_*.

Each layer of the cascaded metasurfaces is a planar array of the hexagonal unit cell (Figure 1b), which comprises an amorphous silicon cylinder patterned on a fused silica substrate. In this work, the lattice constant *P* and cylinder height *H* are fixed to 560 and 600 nm, respectively, for the operation at a wavelength of 1064 nm (*λ*_0_). This high‐aspect‐ratio silicon cylinder supports transverse magnetic (TM), transverse electric (TE), and Fabry‒Perot (FP) modes (Figure 1c). Thus, the overall optical response of a single unit cell is numerically calculated using finite element simulation (Experimental Section and Section S1, Supporting Information) and modeled by temporal coupled mode theory (TCMT) by considering the coupling effect of the modes (Section S2, Supporting Information). The results demonstrate that, by changing the cylinder diameter *D*, the phase of the transmitted light can be modulated from ‐*π* to *π* while keeping the transmission amplitude close to one (Figure 1d,e). This allows the realization of any desired phase profile by unit cells with planarly varied *D*s, forming a light manipulation metasurface.

**Figure 2:**
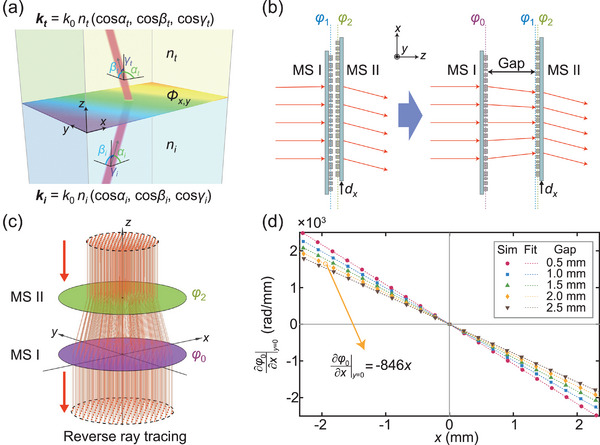
Phase profile design of the cascaded metasurfaces. a) Illustration of the generalized Snell's law of refraction in full space. A metasurface with transmission phase profile *Φ*(*x*, *y*) deflects a wave at the interface of two isotropic homogeneous media with refractive indices *n_i_* and *n_t_*. *Φ*(*x*, *y*) is a binary function of Cartesian coordinates *x* and *y* on the metasurface plane. The incident and outgoing wave vectors are *k_i_* and ***k**_t_*, respectively. The rainbow color gradient represents the local phase profile of the metasurface. b) Design strategies for the cascaded metasurfaces when MS I and MS II are sufficiently close (left) or separated by a relatively large gap (right). c) Illustration of a reverse ray‐tracing simulation to calculate phase profile *φ*_0_ when a constant gap separates MS I and MS II. d) Reverse ray‐tracing calculated points (Sim) and linear fitted lines (Fit) of the partial derivative of phase profile *φ*_0_ with respect to *x* along the *x*‐axis at different gap values for *p* = *q* = –600 (mm^−2^), where *p* and *q* are scale factors of the quadratic terms of phase profiles *φ*_1_ and *φ*_2_.

The behavior of light passing through a single metasurface layer is determined by the generalized Snell's law of refraction,^[^ ^14^ ^]^ which can be described in full space (**Figure** **2**a) as:

(1) $$n_{t}\;\cos\alpha_{t}=n_{i}\;\cos\alpha_{i}+\frac{\lambda_{0}}{2\pi }\frac{\partial \Phi }{\partial x}$$

(2) $$n_{t}\;\cos\beta_{t}=n_{i}\;\cos\beta_{i}+\frac{\lambda_{0}}{2\pi }\frac{\partial \Phi }{\partial y}$$

where *n_i_* and *n_t_* are the refractive indices of the isotropic homogeneous media on the incident and outgoing sides of a metasurface; the metasurface has a transmission phase profile defined as a binary function of Cartesian coordinates *x*, *y* according to *Φ*(*x*, *y*); the incident and outgoing wave vectors are ***k_i_*** = *k*_0_ *n_i_* (cos *α_i_*, cos *β_i_*, cos *γ_i_*) and ***k_t_*** = *k*_0_ *n_t_* (cos *α_t_*, cos *β_t_*, cos *γ_t_*), respectively. The derivation is detailed in Section S3 (Supporting Information).

When the two metasurfaces are placed close enough to each other that the light diffraction within the gap can be omitted (Figure 2b, left column), their phase profiles can be directly added, as indicated by the previous works.^[^ ^22^, ^23^, ^24^, ^25^, ^26^, ^27^, ^31^, ^32^, ^33^, ^34^ ^]^ In other words, supposing that MS I and MS II have phase profiles *φ*_1_(*x*, *y*) and *φ*_2_(*x*, *y*), respectively, the cascaded metasurfaces can be considered as one metasurface with phase profile *Φ*(*x*, *y*) = *φ*_1_(*x*, *y*) + *φ*_2_(*x*‐*d_x_*, *y*‐*d_y_*) when MS II is laterally translated by *d_x_* and *d_y_*. For cascaded metasurface design in this work, we assume that *n_i_* = *n_t_* = 1, ***k_i_*** = ***k*_I_** = *k*_0_ (0, 0, 1), and ***k_t_*** = ***k*_II_** = *k*_0_ (cos [*α*(*d_x_*)], cos [*β*(*d_y_*)], cos [*γ*(*d_x_*, *d_y_*)]) without loss of generality, representing a normally incident plane wave and a dynamically deflected outgoing plane wave. Applying these relations to Equations (1) and (2), we obtain one possible solution of *φ*_1_, *φ*_2_, *α*, and *β* for the dynamic beam deflection:

(3) $$\varphi_{1}\left(x,y\right)=\;-\varphi_{2}\left(x,y\right)=\;px^{2}+qy^{2}$$

(4) $$\alpha \left(d_{x}\right)=\;\arccos\left(\frac{p\lambda_{0}}{\pi }d_{x}\right)$$

(5) $$\beta \left(d_{y}\right)=\;\arccos\left(\frac{q\lambda_{0}}{\pi }d_{y}\right)$$

where *p* and *q* are real numbers that can be arbitrarily chosen to determine the effect of relative translations *d_x_* and *d_y_* on the corresponding direction angles *α* and *β*. The derivation of these equations is detailed in Section S4 (Supporting Information). Note that *α*(0) = *β*(0) = 90°, which means that the light proceeds in the original direction if MS II is not translated. However, as previously described, an ideal plane wave output requires the gap to be infinitely small (without considering near‐field coupling) or equal to the Talbot length^[^ ^40^ ^]^ (calculated to be 0.59 µm for our specific design), whereas an oversized gap will result in unwanted distortion and divergence of the output wavefront.

Since the above design with a small gap between metasurface layers is unrealistic for most practical scenarios, we propose a feasible design paradigm to implement exactly the same tunability with MS II placed away from MS I by a relatively large gap, as shown in Figure 2b (right column). Now, MS I has a new phase profile *φ*_0_, which allows the light to have a phase distribution of precisely *φ*_1_ when arriving at MS II. In this way, the phase of the output wave is still accurately determined by the direct mathematical addition of *φ*_1_(*x*, *y*) and *φ*_2_(*x*‐*d_x_*, *y*‐*d_y_*). The remaining problem is how to calculate *φ*_0_ for a known *φ*_1_ at a prespecified gap size, which may be addressed using the reverse ray‐tracing technique based on the generalized Snell's law of refraction in full space (Equations (1) and (2)), as illustrated in Figure 2c. Due to the principle of reciprocity, when MS II is translated by *d_x_* and *d_y_*, if the light is incident against the direction of the corresponding ***k*_II_**, the light passing through MS II has a phase distribution *φ*_1_. Then, the light propagates across the gap and reaches MS I. If we force the outgoing light from MS I propagate along –***k*_I_**, *φ*_0_ can be calculated by comparing the phase distributions of light on each side of MS I. Thus, *φ*_0_ follows from the phase profile design by means of two considerations: the first is the desired *φ*_1_, and the second is the compensation of the nonuniformity in the phase distortion caused by light propagation across the gap. More details are presented in the Experimental section. It should be noted that the phase addition for the cascaded metasurface device is only validated for unit cell design with insensitivity to a range of oblique incident angles. This discussion is included in Section S1 (Supporting Information).

As an example to demonstrate the reverse ray‐tracing process, without loss of generality, we choose coefficients *p* = *q* = –600 (mm^−2^) to achieve tuning ranges of ∼90° of the direction angles *α* and *β* for translations *d_x_* and *d_y_* within ±3.5 mm. In this case, the effect of *φ*_1_ and *φ*_2_ on light is similar to that of thin lenses under the paraxial approximation. The reverse ray‐tracing calculation provides discrete values of the partial derivatives of *φ*_0_. Due to the circular symmetry of *φ*_1_, we need to consider only ${\frac{\partial \varphi_{o}}{\partial x}|}_{y\;=\;0}$, as shown in Figure 2d and Section S5 (Supporting Information). The good linearity of these discrete points for *x* within ±2 mm indicates that *φ*_0_ may be expressed as a quadratic function. The absolute value of the scale factor of the quadratic term increases as the prespecified gap size decreases, and *φ*_0_ approaches *φ*_1_ since the amount of phase distortion to be compensated is getting smaller. Although we can arbitrarily choose the gap size and calculate a corresponding *φ*_0_, the cascaded metasurface device is set to operate at a gap of 2 mm for further demonstration. In this case, the linear fitting (Figure 2d) and integration lead to *φ*_0_(*x*,*y*) = −423(*x* ^2^ + *y* ^2^). By this design, at the preset gap size, the cascaded MS I (*φ*_0_) and MS II (*φ*_2_) are expected to dynamically steer the optical wave without distorting the output plane wavefront, which is advantageous for many applications where the divergence is required to be as small as possible. More details about the designed phase profiles are presented in Section S6 (Supporting Information). The operating principle of the cascaded metasurface device can be intuitively interpreted by the lens design approach, as illustrated in Section S7 (Supporting Information).

### 2.2 Beam‐Steering Performance

**Figure 3:**
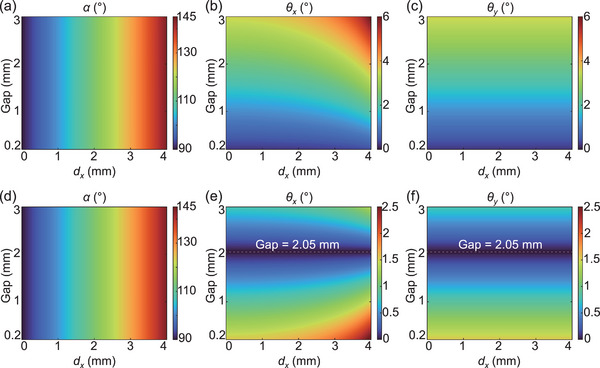
Forward ray‐tracing simulation results of the cascaded metasurfaces. a–c) Direction angle *α* and divergence angles *θ_x_* and *θ_y_* when MS I and MS II have phase profiles *φ*_1_ and *φ*_2_. d–f) Direction angle *α* and divergence angles *θ_x_* and *θ_y_* when MS I and MS II have phase profiles *φ*_0_ and *φ*_2_.

To validate the reverse ray‐tracing approach for obtaining *φ*_0_, forward ray‐tracing simulations are utilized to evaluate the tunable responses of cascaded metasurfaces with different phase profiles. The results are shown in **Figure** **3** and Movie S1 (Supporting Information). Due to the circular symmetry of MS I and MS II, we need to consider only the case where MS II moves along the *x*‐axis. For these simulations, MS I is normally incident on its center by a group of parallel light rays with a circular cross‐section of a 0.5‐mm radius. The direction angle *α* is calculated as the average of the *x* direction angles of the outgoing light rays, and the divergence angles *θ_x_* (*θ_y_*) are calculated as half of the maximum *x* (*y*) direction angle difference in the outgoing light rays. The colormaps in Figure 3 exhibit the simulation results for two cascaded metasurface setups: MS I and MS II with phase profiles *φ*_1_ and *φ*_2_ (the top row); MS I and MS II with phase profiles *φ*_0_ and *φ*_2_ (the bottom row). For both cases, *α* is observed to be independent of the gap size, and the simulated values agree with those calculated through Equation (4). For the cascaded *φ*_1_ (MS I) and *φ*_2_ (MS II), *θ_x_* and *θ_y_* rapidly grow with the increasing gap size. Even a small gap size of 0.2 mm introduces divergence greater than 0.2°. For the cascaded metasurfaces with *φ*_0_ and *φ*_2_, an optimal gap of 2.05 mm, which deviates from the design value of 2 mm due to fitting error, leads to near‐zero divergence. At this optimal gap size, both *θ_x_* and *θ_y_* are consistently less than 0.007° as *d_x_* increases from 0 to 4 mm.

**Figure 4:**
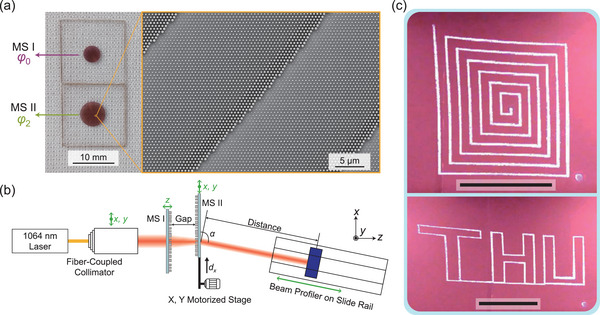
Fabricated cascaded metasurfaces and characterization setup. a) Images of MS I with phase profile *φ*_0_ and MS II with phase profile *φ*_2_. The aperture diameters of MS I and MS II are 4 and 6 mm, respectively. The image on the left was taken with the two metasurfaces placed on a regular cleanroom wipe, and the microscopic inset image was obtained by scanning electron microscopy (SEM). b) Schematic of the experimental setup for characterization of beam‐steering performance. The green arrows represent the degrees of freedom of movement of the corresponding elements. c) Tracks of a deflected light spot on a wall 3 m away as MS II are translated along certain curves. MS I and MS II have phase profiles *φ*_0_ (MS I) and *φ*_2_ (MS II), and the gap between them is 2.12 mm. Scale bars: 50 cm.

To experimentally demonstrate the concept of cascaded metasurfaces, we fabricate three metasurfaces with phase profiles *φ*_0_ *, φ*_1_, and *φ*_2_ and diameters of 4, 4, and 6 mm, respectively, as detailed in the Experimental Section. The diameters of the metasurfaces are determined by the ability of the unit cells to discretely sample the phase profiles, which is detailed in Section S8 (Supporting Information). The three metasurfaces can realize two cascading setups: MS I and MS II with phase profiles *φ*_1_ and *φ*_2_; MS I and MS II with phase profiles *φ*_0_ and *φ*_2_ (**Figure** **4**a). The experimental setup for the characterization of beam‐steering performance is depicted in Figure 4b. In this system, a fiber‐coupled collimator is mounted on a manual translational stage to ensure that the light beam is incident on the center of MS I. Due to the circular symmetry of MS I and MS II, full characterization of the cascaded metasurfaces can be obtained by translating MS II only along the *x* direction. We use the full‐width at half‐maximum (FWHM) diameter to represent the size of the Gaussian beam to avoid interference from unwanted background or stray light. The divergence angles are obtained based on the deflected beam diameters measured by the beam profiler at different distances. More details about the experiments are presented in the Experimental Section.

To qualitatively evaluate the continuous 2D beam‐steering capabilities, *φ*_0_ (MS I) and *φ*_2_ (MS II) are cascaded, and the gap size is adjusted to its optimal value of 2.12 mm for the smallest deflected beam divergence. Then, MS II is translated along predetermined curves. At the same time, the tracks of the deflected light spot on a wall 3 m away from the metasurface device are recorded with a near‐infrared CCD camera (wavelength range: 700–1100 nm), as shown in Figure 4c. The complete 2D beam‐steering process is demonstrated in Movie S2 (Supporting Information).

**Figure 5:**
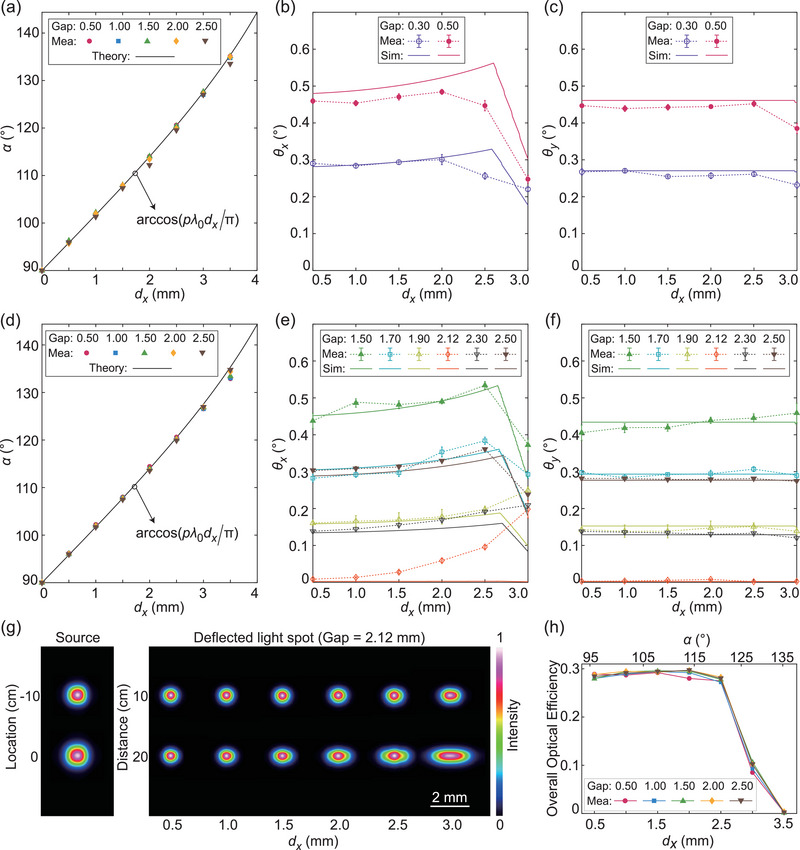
Characterization of the cascaded metasurfaces. a–c) Measured (Mea) and calculated direction angles *α* and divergence angles *θ_x_* and *θ_y_* for cascaded *φ*_1_ (MS I) and *φ*_2_ (MS II) for multiple gap sizes (mm). d–f) Measured (Mea) and calculated direction angle *α* and divergence angles *θ_x_* and *θ_y_* for cascaded *φ*_0_ (MS I) and *φ*_2_ (MS II) for multiple gap sizes (mm). The calculation results are obtained either by Equation (4) (Theory) or forward ray‐tracing simulations (Sim). g) Intensity profiles of the incident (left) and deflected (right) beams for cascaded *φ*_0_ (MS I) and *φ*_2_ (MS II) at the gap of 2.12 mm. The incident beam profiles are measured at 10 cm before the light arrives at MS I (top) and at the location of MS I (bottom). The deflected beam profiles are measured 10 and 20 cm beyond MS II. h) Overall optical efficiency measurement results for cascaded *φ*_0_ (MS I) and *φ*_2_ (MS II) as a function of *d_x_* (*α*) at multiple gap sizes (mm).

Next, the overall performance of the cascaded metasurfaces is characterized by measuring the direction angle *α*, divergence angles *θ_x_* and *θ_y_*, and optical efficiency at multiple gap sizes. As shown in **Figure** **5**, tunable responses of both aforementioned two cascading setups are evaluated: MS I and MS II with phase profiles *φ*_1_ and *φ*_2_ (the top row); MS I and MS II with phase profiles *φ*_0_ and *φ*_2_ (the middle and bottom rows). In both cases, the direction angle *α* demonstrates a large tunable range of ∼45° for *d_x_* within 0–3.5 mm, and the measurement results agree well with the theoretical predictions (Figure 5a,d). In the design stage, a smaller metasurface lattice constant *P* can be adopted to achieve larger tunable ranges of *α* and *β*, as analyzed in Section S9 (Supporting Information). For cascaded *φ*_1_ (MS I) and *φ*_2_ (MS II), the divergence of the deflected light beam increases as the gap increases (Figure 5b,c). The gap size of 0.3 mm, which is relatively small compared with the metasurface sizes, leads to rather large *θ_x_* and *θ_y_* of ∼0.3°. We measured *θ_x_* and *θ_y_* up to a maximum gap size of 0.5 mm because the size of the light spot exceeds the detection area of the beam profiler when placed further away from the beam waist at larger gaps. For cascaded *φ*_0_ (MS I) and *φ*_2_ (MS II), the divergence of the deflected light beam decreases and then increases as the gap increases (Figure 5e,f), and the minimal divergence angle values appear at the gap of 2.12 mm. These results demonstrate that our design paradigm substantially reduces the implementation difficulties of cascaded metasurfaces by allowing them to operate optimally at large prespecified gap sizes that are easy to keep and control.

Measured intensity profiles of the incident beam (Figure 5g, left) and the sizes of fabricated metasurfaces are used to perform another round of forward ray‐tracing simulations, as shown in Section S10 and Movie S3 (Supporting Information). The results are plotted as solid lines in Figure 5b,c,e,f. Due to the finite diameters of MS I and MS II, the cascaded metasurfaces have a decreasing effective aperture size as *d_x_* increases. At large *d_x_* values, part of the light reaching MS II plane lies outside the effective metasurface region, which reduces the deflected beam size and divergence angle values, as illustrated in Section S11 (Supporting Information). Besides misalignment between metasurfaces and measurement errors, two causes may attribute to the deviations between the simulated and measured values. First, our ray‐tracing model omits the energy distribution of the real Gaussian light source for the sake of simplicity: we consider only a group of uniform geometric rays whose boundary is the FWHM profile of the real incident beam. Therefore, in experiments, the effect from a reduction in effective aperture occurs at a smaller *d_x_* than in simulations. Second, the ray‐tracing scheme ignores the wave nature of light. In simulations, the rays out of the metasurface apertures straightforwardly travel along their original directions. In experiments, diffraction and scattering at the edges of metasurfaces may introduce stray light and extra beam divergence.

Note that the deviations between simulated and measured values of *θ_x_* (Figure 5b,e) are larger than those of *θ_y_* (Figure 5c,f), especially at large *d_x_* values. This is because, in the experiments, the effective aperture size in the *x* direction decreases as *d_x_* increases, while the effective aperture size in the *y* direction remains almost constant and large enough to cover the light beam size. Accordingly, the two causes mentioned in the last paragraph have much greater effects on *θ_x_* than on *θ_y_*. To obtain better beam‐steering performance in experiments and applications, we may simply adopt a smaller incident beam size and larger metasurface diameters.

To evaluate the uniformity of the tuning process, the overall optical efficiency is measured for cascaded *φ*_0_ (MS I) and *φ*_2_ (MS II) at multiple gap values (Figure 5h). Here, we define the overall optical efficiency as the ratio of the power of the deflected beam to that of the incident beam. As *d_x_* (or *α*) increases, the magnitude of the overall optical efficiency remains essentially constant at around 28% until it is affected by the reduction in effective aperture size, which is favorable for many practical applications. When *d_x_* reaches 3 mm (*α* reaches ∼125°), the edge of MS II exactly aligns with the center of MS I across the gap, and the efficiency value is reduced by approximately half, as expected. Measurement results of the transmission efficiency and the deflection efficiency are presented in the Section S12 (Supporting Information). The side mode suppression ratio (SMSR), defined as the intensity ratio between the desired main lobe and the highest side lobe,^[^ ^47^ ^]^ is simulated as detailed in Section S13 (Supporting Information). The simulated SMSR is around –4 dB. The SMSR and deflection efficiency may be improved by decreasing the periodicity of the metasurface unit cell and perform systematic optimization of the overall cascaded metasurfaces.^[^ ^48^ ^]^

## 3 Discussion

The designed and fabricated cascaded metasurface device in this paper demonstrates a versatile 2D beam‐steering platform realized by artificially engineered metasurfaces. Since the incident wavevector ***k*_I_** can be arbitrarily chosen in the design, the platform is compatible with various types of light sources without increasing the number of metasurfaces or other optical elements. For example, we may slightly modify phase profile *φ*_0_ to make the cascaded metasurfaces suitable for steering the light from a point source, as shown in Movie S4 (Supporting Information). Compared with beam‐steering methods based on optical phase arrays^[^ ^6^ ^]^ and focal plane switch arrays,^[^ ^7^ ^]^ this platform works as passive optical elements and is able to transmit light beams of much higher optical power. Compared with rotational microelectromechanical‐based mirrors,^[^ ^4^, ^5^ ^]^ this platform features a larger steering range and easier controlled mechanical motion form. Compared with electrically tunable metasurface phase arrays,^[^ ^17^, ^49^ ^]^ our proposed platform exhibits limited beam‐steering speed, but it possesses higher optical efficiency, a broader tuning range and better output beam quality. Therefore, the proposed platform exhibits great promise for implementing of semisolid and highly compact beam‐steering systems for applications such as LiDAR and optical communication.

Moreover, the proposed design paradigm incorporating a numerical unit cell model and a theoretical ray‐tracing scheme brings tunable cascaded metasurfaces from theory to experimental demonstration by allowing them to operate under easily achievable optimal conditions. The ray‐tracing scheme alleviates the dependency on computational resources and enables quick design by avoiding iterative or finite element algorithms. The paradigm can be generalized and applied to the cascading of metasurfaces based on other tuning mechanisms such as electrical gating,^[^ ^16^ ^]^ phase‐changing materials,^[^ ^18^ ^]^ and stretchable substrate.^[^ ^20^ ^]^ The paradigm also provides more possibilities for manipulating electromagnetic waves from visible light to millimeter waves, and may inspire more intriguing applications for other artificially engineered materials, for example, acoustic metamaterials.^[^ ^50^ ^]^

## 4 Experimental Section

### Simulation of Metasurfaces

The transmission coefficients and electromagnetic field distributions of the metasurface unit cell were calculated using the finite element simulation software COMSOL Multiphysics. Periodic boundary conditions were set in the *x* and *y* directions to create an infinite periodic array of the unit cell as depicted in Figure 1b. The array was bounded in the *z* direction by air columns with perfectly matched layers (PMLs) at both the input and output interfaces and normally illuminated by a *y*‐polarized plane wave. The fused silica substrate and amorphous silicon cylinders were assumed to be lossless with refractive indices of 1.45 and 3.38, respectively.

### Reverse Ray‐Tracing Technique to Calculate *φ*_0_

In the reverse ray–tracing setup, MS II (with phase profile *φ*_2_) is translated by arbitrary values of *d_x_* and *d_y_* and the incident light rays are set to proceed along the corresponding forward transmission wavevector ‐***k*_II_**. Under such conditions, due to the principle of reciprocity, the light after passing through MS II would exhibit a phase distribution of precisely *φ*_1_. For the sake of simplicity, the authors might set *d_x_* = *d_y_* = 0; thus, ‐***k*_II_** = (0, 0, –1), as shown in Figure 2c. Then, the light rays were propagated through the prespecified gap and arrived at MS I with various angles. To obtain the unknown *φ*_0_, the authors assumed that the light rays traveled along ‐***k*_I_** after passing through MS I. By comparing the directions of light rays on each side of MS I, the partial derivatives of *φ*_0_ could be calculated by slightly modified Equations (1) and (2):

(6) $$\frac{\partial \Phi }{\partial x}=\frac{2\pi }{\lambda_{0}}\;\left(n_{t}\cos\alpha_{t}-n_{i}\cos\alpha_{i}\right)$$

(7) $$\frac{\partial \Phi }{\partial y}=\frac{2\pi }{\lambda_{0}}\;\left(n_{t}\cos\beta_{t}-n_{i}\cos\beta_{i}\right)$$

Note that this reverse ray‐tracing process could provide only discrete values for $\frac{\partial \varphi_{o}}{\partial x}$ and $\frac{\partial \varphi_{o}}{\partial y}$, since the traced light rays were discretely distributed in space. Curve fitting and integration were then performed to obtain a solution of *φ*_0_. The whole reverse ray‐tracing process could be successfully completed as long as all the involved cosine values were in the range of –1 to 1. Both the forward and reverse ray‐tracing algorithms were implemented using MATLAB software.

### Fabrication of the Metasurfaces

Three metasurfaces with different phases (*φ*_0_(*x*,*y*) = −423 × (*x* ^2^ + *y* ^2^), *φ*_1_(*x*,*y*) = −600 × (*x* ^2^ + *y* ^2^), and *φ*_2_(*x*,*y*) = 600 × (*x* ^2^ + *y* ^2^)) were fabricated for experimentation using the same nanofabrication process. A 600‐nm‐thick amorphous silicon layer was deposited on a 500‐µm‐thick fused silica substrate through plasma‐enhanced chemical vapor deposition (PECVD). The intended metasurface pattern was then created by electron beam lithography (EBL) on a positive‐tone photoresist (ZEP520A, Zeon Chemicals). Next, a 50‐nm Cr layer was produced as a hard mask by electron beam evaporation and a lift‐off process. Finally, nanocylinders were generated by inductively coupled plasma (ICP) etching.

### Experimental Characterization of the Beam‐Steering Performance

A beam profiler (BP209IR1/M, Thorlabs) was mounted on a motorized straight slide rail that could be manually moved on the optical stage to bring it in alignment with the direction of light propagation. A near‐infrared CCD camera (700–1100 nm) was used to locate the outgoing beam spot. The intensity profile of the 1064 nm laser (CoLID‐I‐1064, Connet Laser Technology) was measured at two locations 10 cm apart to evaluate the collimation of the beam. Then, MS I was placed at the location further from the light source, and thus, the measurement results from this location provided necessary information about the beam incident on MS I in the reported experiments. These properties of the source were shown in Figure 5g (left) and in Section S10 (Supporting Information). The fiber‐coupled collimator (GCX‐LF11APC‐1064, Daheng Optics) was translated along the *x* and *y* directions by a manual stage to ensure that the light was incident on the center of MS I.

The gap between cascaded metasurfaces was controlled by translating MS I with another manual stage along the *z* direction. MS II was driven by a motorized stage along the +*x* direction to deflect the beam, as depicted in Figure 4b. The outgoing direction angle was obtained through the coordinates of the deflected beam spot center on the light screen, as captured by the CCD camera. The collimation of the deflected beam was obtained based on the intensity profiles measured at two locations 10 cm apart, both of which were away from the beam waist. The optical power of the deflected beam was measured by an optical power meter (PM400, Thorlabs).

The collimation of the incident and deflected beam was quantified by divergence angles along both *x*‐ and *y*‐ axes. In every measurement, the FWHM beam diameters were recorded for 1 min to minimize the random errors. The divergence angles were calculated as:

(8) $$\theta_{x}=\tan^{-1}\left|\frac{\bar{D_{x1}}-\bar{D_{x2}}}{2S}\right|\;$$

(9) $$\theta_{y}=\tan^{-1}\left|\frac{\bar{D_{y1}}-\bar{D_{y2}}}{2S}\right|\;$$

where ($\bar{D_{x1}}$ and $\bar{D_{x2}}$) and ($\bar{D_{y1}}$ and $\bar{D_{y2}}$) are the average FWHM beam diameters along the *x* and *y* direction at the two measurement locations, respectively, and *S* = 10 cm is the distance between those locations.

## Conflict of Interest

The authors declare no conflict of interest.

## Supporting information

Supporting Information

Supporting Information Movie S1

Supporting Information Movie S2

Supporting Information Movie S3

Supporting Information Movie S4

## Data Availability

The data that support the findings of this study are available from the corresponding author upon reasonable request.
