# Supplementary material for: Highly Tunable Cascaded Metasurfaces for Continuous Two‐Dimensional Beam Steering
Source: Adv Sci (Weinh). 2023 Jun 20;10(24):2300542. doi: 10.1002/advs.202300542 (PMC10460883; doi:10.1002/advs.202300542)
Supplement: Supplementary file 1 — Supporting Information [file ADVS-10-2300542-s004.pdf]

ADVANCED SCIENCE
Open Access

Supporting Information

for *Adv. Sci.*, DOI 10.1002/advs.202300542

Highly Tunable Cascaded Metasurfaces for Continuous Two-Dimensional Beam Steering

Lingyun Zhang, Li Zhang, Rongbo Xie, Yibo Ni, Xiaoyu Wu, Yuanmu Yang, Fei Xing, Xiaoguang Zhao* and Zheng You**

Supporting Information

Highly tunable cascaded metasurfaces for continuous two-dimensional beam steering

Lingyun Zhang, Li Zhang, Rongbo Xie, Yibo Ni, Xiaoyu Wu, Yuanmu Yang, Fei Xing, Xiaoguang Zhao*, Zheng You**

1. Numerical simulation of the electromagnetic responses of the metasurface unit cell.

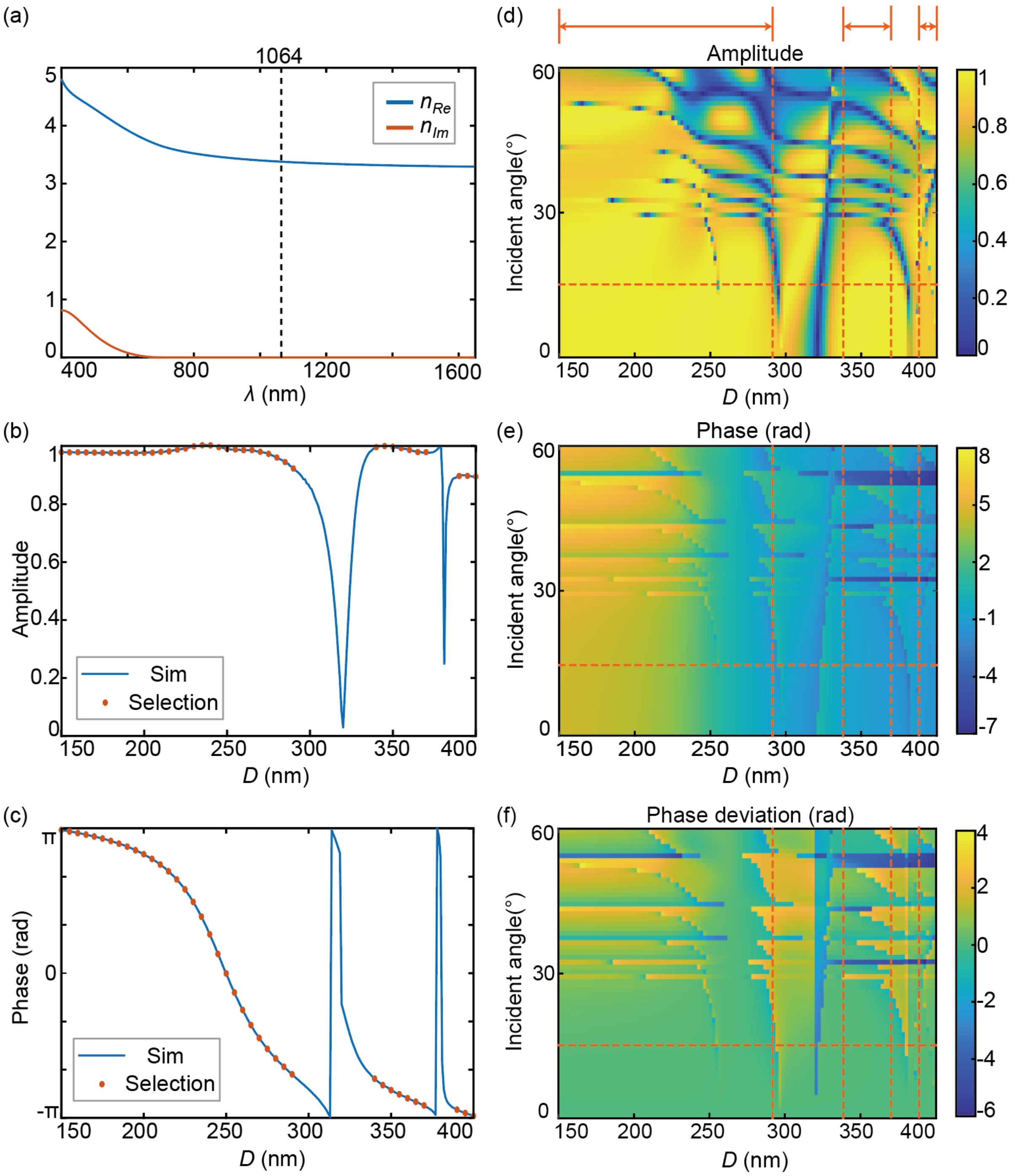

Figure S1. Details about the numerical simulation of the metasurface unit cell with a lattice constant *P* = 560 nm and height *H* = 600 nm. (a) Real part (n_{Re}) and imaginary part (n_{Im}) of the refractive index of the grown amorphous silicon film versus free-space wavelength λ measured by the ellipsometer. (b and c) Simulated amplitude and phase as functions of cylinder diameter *D* (Sim) and selected *D* values for phase profile sampling of the metasurfaces (Selection) under normal incidence at the wavelength of 1064 nm. (d, e and f) The amplitude, phase and phase deviation of the transmission coefficient for different *D*s and diferent oblique

incident angles at the wavelength of 1064 nm. The arrows above (d) indicate regions from which the *D* values are selected for phase profile sampling.

This section provides more details about numerical simulation of the metasurface unit cell.

Before numerical simulation, an amorphous silicon film was deposited on the fused silica substrate through the PECVD (plasma-enhanced chemical vapor deposition) process. Then, the optical property of the film was measured by the ellipsometer, as presented by Figure S1a. The obtained dielectric constants ($\varepsilon_r = N^2 / \mu_r = (n_{Re} + \mathrm{i}n_{Im})^2 / \mu_r$) were utilized to perform the numerical simulation of the metasurface unit cell to generate results in Figure 1c - 1e and Figure S1b - S1f. Specifically, at the working wavelength of 1064 nm, the dielectric constant $\varepsilon_r = (3.38 + \mathrm{i}0)^2 / 1 = 11.42$. The refractive index of the fused silica substrate was provided by the wafer manufacturer. At the wavelength of 1064 nm, the dielectric constant $\varepsilon_r = (1.45 + \mathrm{i}0)^2 / 1 = 2.10$.

A series of cylinder diameter (*D*) values were chosen for phase profile sampling of the metasurfaces, as graphed in Figure S1b and S1c. The unit cells with selected *D* values exhibit high transmission and full 2π phase coverage at 1064 nm. The maximum phase difference between selected neighboring *D* values is calculated to be 24°, resulting in an absolute phase sampling error less than 12°.

To verify the incident-angle-insensitivity of our silicon cylinder unit cell design, we performed additional numerical simulation and the results are presented in Figure S1d and S1e. Furthermore, the phase deviation of oblique incidence to normal incidence for is calculated, as presented in Figure S1f. It is observed that the selected unit cells exhibit high transmission and negligible phase deviations when the incident angle is less than 15°.

2. Derivation of the three-mode two-port TCMT model.

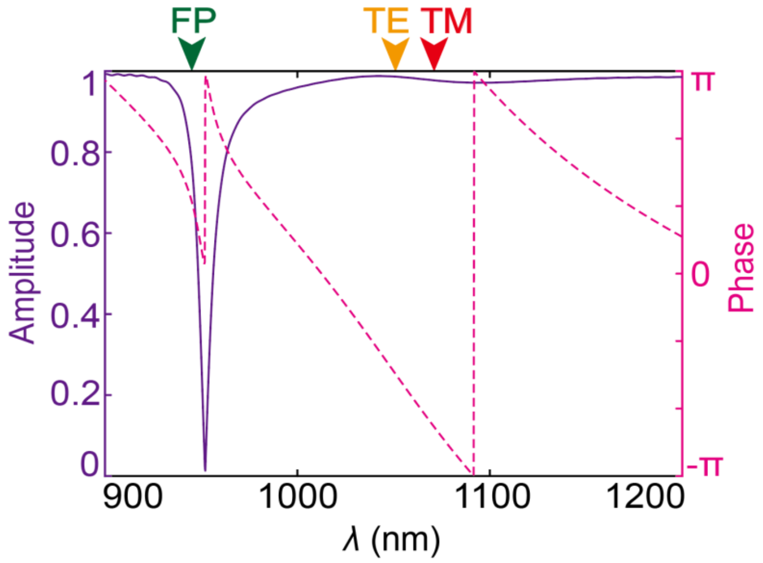

Figure S2. Simulated transmission amplitude and phase of the metasurface unit cell with a lattice constant *P* = 560 nm, diameter *D* = 250 nm and height *H* = 600 nm. The transverse magnetic (TM), transverse electric (TE), and Fabry–Perot (FP) resonance modes occur at the free-space wavelength of 1071, 1051 and 945 nm, respectively.

The coupling of transverse magnetic (TM), transverse electric (TE), and Fabry–Perot (FP) modes (Figure 1c and Figure S2) is investigated by the three-mode two-port temporal coupled mode theory (TCMT) model[51-52], which can be expressed as:

$$\frac{\mathrm{d}\boldsymbol{a}}{\mathrm{d}t} = (j\Omega - \Gamma)\boldsymbol{a} + K\boldsymbol{s}_+ \tag{S1}$$

$$\boldsymbol{s}_- = C\boldsymbol{s}_+ + D\boldsymbol{a} \tag{S2}$$

where $\boldsymbol{a}$ is the vector composed of the amplitudes of each resonance mode, $\boldsymbol{s}_+$ and $\boldsymbol{s}_-$ composed of the input and output amplitudes of each port, Ω is the matrix of the resonance frequencies and the direct coupling coefficients, K and D are the matrixes of the coupling coefficients between resonances and input and output ports, Γ is the matrix of the decay rates and the indirect coupling coefficients. Assuming that the system is lossless with mirror plane symmetry and that the three modes are in the order even-odd-even, we further define the matrices as:

$$C = \begin{bmatrix} 0 & 1 \\ 1 & 0 \end{bmatrix}, \boldsymbol{s}_+ = \begin{bmatrix} s_{1+} \\ s_{2+} \end{bmatrix}, \boldsymbol{s}_- = \begin{bmatrix} s_{1-} \\ s_{2-} \end{bmatrix}, \boldsymbol{a} = \begin{bmatrix} a_1 \\ a_2 \\ a_3 \end{bmatrix} \tag{S3}$$

$$D = K = \begin{bmatrix} \sqrt{2\gamma_{e1}}e^{j\frac{\pi}{2}} & \sqrt{2\gamma_{e2}} & \sqrt{2\gamma_{e3}}e^{j\frac{\pi}{2}} \\ \sqrt{2\gamma_{e1}}e^{j\frac{\pi}{2}} & \sqrt{2\gamma_{e2}}e^{j\pi} & \sqrt{2\gamma_{e3}}e^{j\frac{\pi}{2}} \end{bmatrix} \tag{S4}$$

$$\Omega = \begin{bmatrix} \omega_{01} & 0 & \kappa \\ 0 & \omega_{02} & 0 \\ \kappa & 0 & \omega_{03} \end{bmatrix} \tag{S5}$$

$$\Gamma = \frac{1}{2}D^{*}D \tag{S6}$$

where κ is the direct coupling coefficient between the two even modes and γei is the radiation decay rate of mode i. To simplify the model, κ is set to 0. Substituting equations S3-S6 into S1 and S2, we obtain the reflection and transmission coefficients r and t of the system:

$$\begin{bmatrix} r \\ t \end{bmatrix} = C\begin{bmatrix} 1 \\ 0 \end{bmatrix} - D(j(\Omega - I\omega) - \Gamma)^{-1}K^{T}\begin{bmatrix} 1 \\ 0 \end{bmatrix} \tag{S7}$$

According to Equation S7, we fit the spectrum for *D* = 150–400 nm with a sampling spacing of 50 nm and obtain six sets of TCMT parameters. Finally, we perform cubic spline interpolation for the obtained TCMT parameters and substitute them into Equation S7 to obtain the theoretical results of the three-mode two-port TCMT model shown in Figure 1d, e. These calculation results agree well with the finite element simulated curves, demonstrating that the 2π phase coverage and high transmission amplitude of the high-aspect-ratio silicon cylinder are attributed to the well-engineered coupling of the three resonance modes and low material loss.

3. Derivation of the generalized Snell's law of refraction in full space.

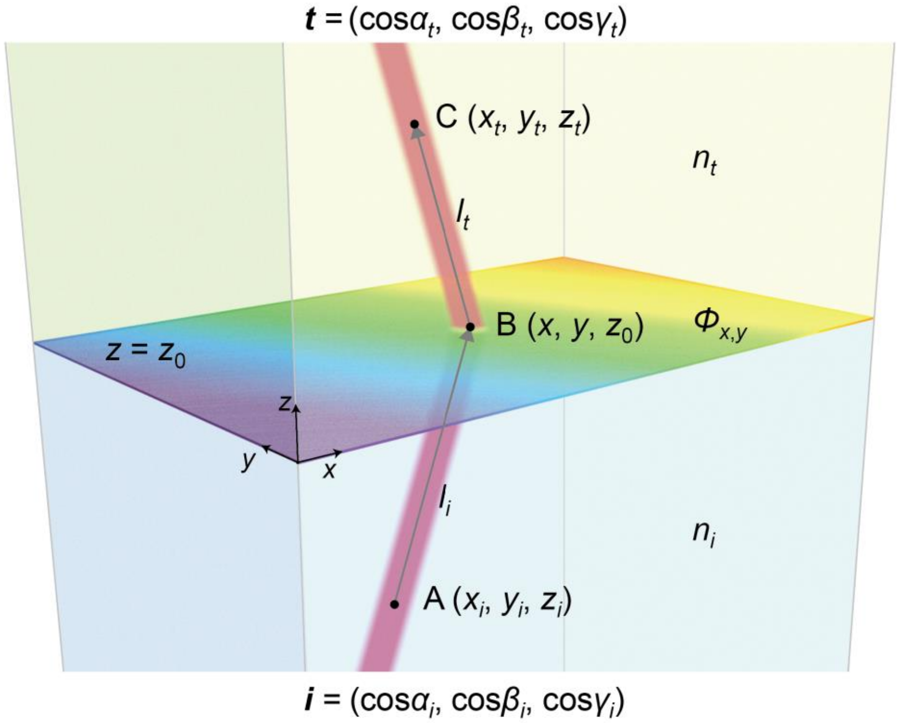

Figure S3 | Schematic used to derive the generalized Snell's law of refraction in full space. A (x_i, y_i, z_i), B (x, y, z_0) and C (x_t, y_t, z_t) are three points defining a feasible light propagation path. Point A and C are located in the isotropic homogenous media with refractive indices n_i and n_t, respectively. Point B is located on the metasurface interface with a phase profile $\Phi(x, y)$.

As shown in Figure S3, the spatial coordinates of points A, B and C are (x_i, y_i, z_i), (x, y, z_0) and (x_t, y_t, z_t), respectively. Point B is located on the transmissive metasurface ($z = z_0$). Assuming that A-B-C is a light propagation path, the optical path length (L) is:

$$L(x,y) = n_i l_i + \frac{\lambda_0}{2\pi}\Phi(x,y) + n_t l_t \tag{S8}$$

where li and lt are the lengths of vectors $\overrightarrow{AB}$ and $\overrightarrow{BC}$:

$$l_i = \sqrt{(x-x_i)^2 + (y-y_i)^2 + (z_0-z_i)^2} \tag{S9}$$

$$l_t = \sqrt{(x_t-x)^2 + (y_t-y)^2 + (z_t-z_0)^2} \tag{S10}$$

According to Fermat's principle, the incident and transmitted waves should satisfy the following equations:

$$\begin{aligned}\frac{\partial L}{\partial x} &= n_i \frac{x - x_i}{l_i} - n_t \frac{x_t - x}{l_t} + \frac{\lambda_0}{2\pi}\frac{\partial \Phi}{\partial x} \\ &= n_i \cos\alpha_i - n_t \cos\alpha_t + \frac{\lambda_0}{2\pi}\frac{\partial \Phi}{\partial x} \\ &= 0\end{aligned} \tag{S11}$$

$$\begin{aligned}\frac{\partial L}{\partial y} &= n_i \frac{y - y_i}{l_i} - n_t \frac{y_t - y}{l_t} + \frac{\lambda_0}{2\pi}\frac{\partial \Phi}{\partial y} \\ &= n_i \cos\beta_i - n_t \cos\beta_t + \frac{\lambda_0}{2\pi}\frac{\partial \Phi}{\partial y} \\ &= 0\end{aligned} \tag{S12}$$

Hence, the generalized Snell's law of refraction in full space can be expressed as:

$$n_t \cos\alpha_t = n_i \cos\alpha_i + \frac{\lambda_0}{2\pi}\frac{\partial \Phi}{\partial x} \tag{S13}$$

$$n_t \cos\beta_t = n_i \cos\beta_i + \frac{\lambda_0}{2\pi}\frac{\partial \Phi}{\partial y} \tag{S14}$$

4. Design of phase profiles $\varphi_1(x, y)$ and $\varphi_2(x, y)$.

When the gap between MS I and MS II is sufficiently small, we obtain:

$$\begin{aligned}\frac{\partial \Phi(x,y)}{\partial x} &= \frac{\partial\left(\varphi_1(x,y)+\varphi_2(x-d_x,y-d_y)\right)}{\partial x}\\ &= \frac{2\pi}{\lambda_0}\cos[\alpha(d_x)]\\ &= 2px - 2p\left(x - \frac{\pi}{p\lambda_0}\cos[\alpha(d_x)]\right)\end{aligned} \quad \text{(S15)}$$

$$\begin{aligned}\frac{\partial \Phi(x,y)}{\partial y} &= \frac{\partial\left(\varphi_1(x,y)+\varphi_2(x-d_x,y-d_y)\right)}{\partial y}\\ &= \frac{2\pi}{\lambda_0}\cos[\beta(d_y)]\\ &= 2qy - 2q\left(y - \frac{\pi}{q\lambda_0}\cos[\beta(d_y)]\right)\end{aligned} \quad \text{(S16)}$$

where p and q are real numbers. Assuming that:

$$\frac{\partial\varphi_1(x,y)}{\partial x} = -\frac{\partial\varphi_2(x,y)}{\partial x} = \frac{2\pi}{\lambda_0}(2px) \quad \text{(S17)}$$

$$\frac{\partial\varphi_1(x,y)}{\partial y} = -\frac{\partial\varphi_2(x,y)}{\partial y} = \frac{2\pi}{\lambda_0}(2qy) \quad \text{(S18)}$$

$$d_x = \frac{\pi}{p\lambda_0}\cos[\alpha(d_x)] \quad \text{(S19)}$$

$$d_y = \frac{\pi}{q\lambda_0}\cos[\beta(d_y)] \quad \text{(S20)}$$

Then, one possible solution of φ_1, φ_2, α and β can be expressed as:

$$\varphi_1(x,y) = -\varphi_2(x,y) = px^2 + qy^2 \quad \text{(S21)}$$

$$\alpha(d_x) = \arccos\left(\frac{p\lambda_0}{\pi}d_x\right) \quad \text{(S22)}$$

$$\beta(d_y) = \arccos\left(\frac{q\lambda_0}{\pi}d_y\right) \quad \text{(S23)}$$

5. Calculation of the partial derivatives of φ_0.

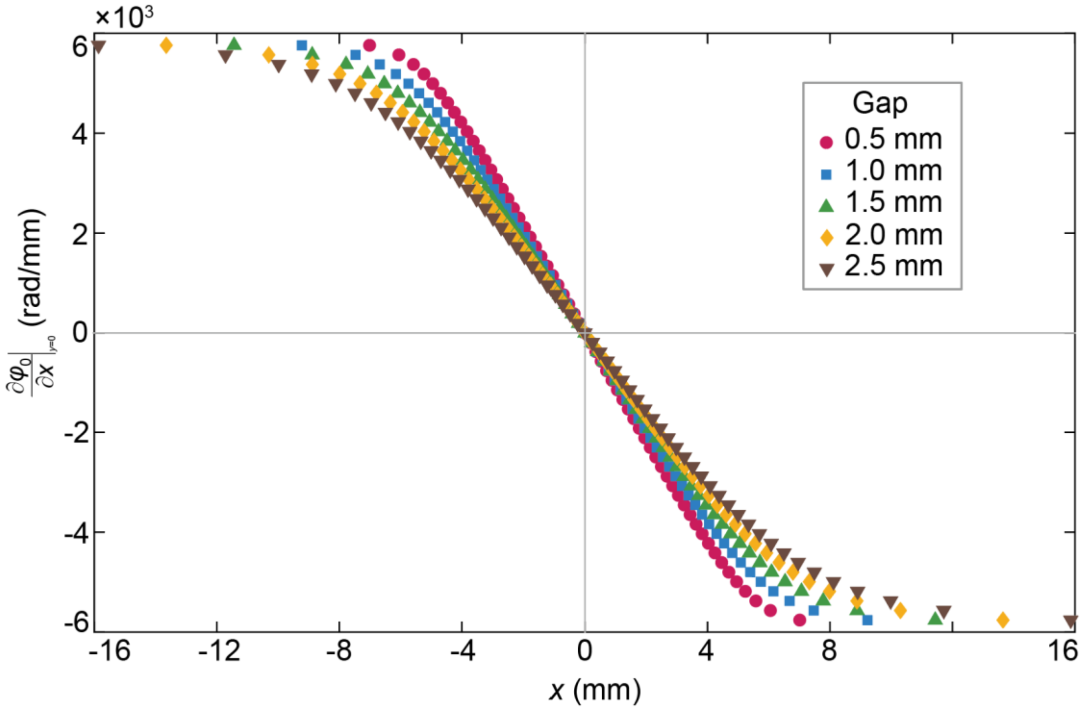

Figure S4 | Calculation results of the partial derivatives of φ_0 along the *x*-axis.

Due to the circular symmetry of the metasurface device layers, we need to consider the partial derivatives only along the *x*-axis ($\frac{\partial \varphi_o}{\partial x}\big|_{y=0}$ and $\frac{\partial \varphi_o}{\partial y}\big|_{y=0}$), where $\frac{\partial \varphi_o}{\partial y}\big|_{y=0} \equiv 0$. $\frac{\partial \varphi_o}{\partial x}\big|_{y=0}$ is calculated by comparing the directions of light rays on each side of MS I according to the reverse ray-tracing setup, and the results are plotted in Figure S4. A nonlinear function is needed to fit the points in the figure. However, there is an upper limit of the metasurface radius as described in Section 8 of the Supporting Information and MS I only needs to be large enough to cover the incident light spot. Therefore, we can limit experimentation to an *x* range from -2 mm to 2 mm (Figure 2d). Within this range, a linear function is sufficient to achieve high fitting accuracy.

6. Designed phase profiles and the discrete sampling by metasurface unit cells.

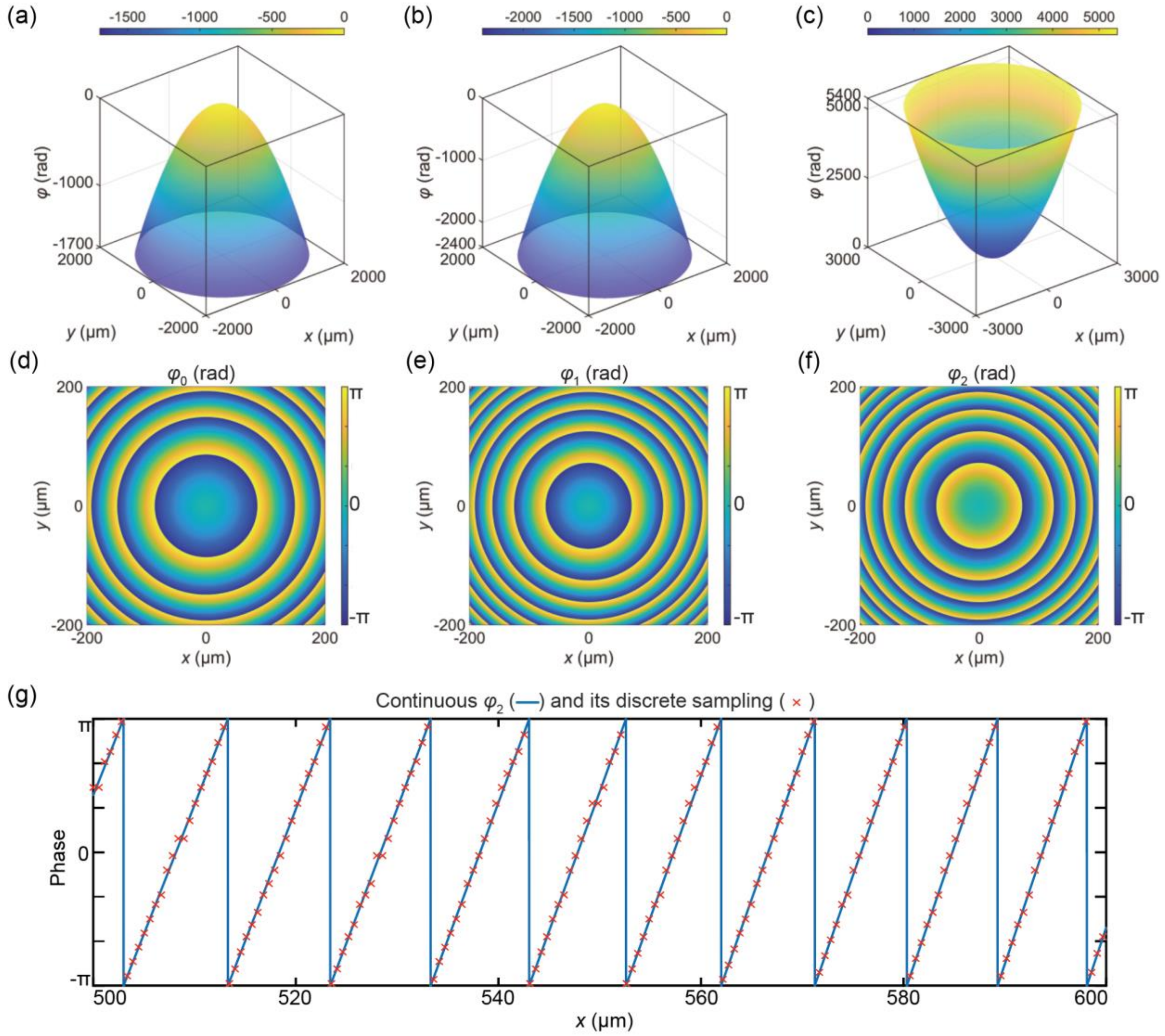

Figure S5 | Designed phase profiles and the discrete sampling by metasurface unit cells. (a, b and c) 3D contour maps of φ_0, φ_1 and φ_2, respectively. (d) (e) (f) 2D contour maps in the central region of the device of φ_0, φ_1 and φ_2 wrapped to [-π, π], respectively. (g) Continuous phase profile φ_2 and its discrete sampling along *x*-axis by unit cells with varying *D*s.

The designed three phase profiles are presented as contour maps shown in Figure S5a – S5f. For implementation of the metasurface device, unit cells with different *D*s (Section 1 of the Supporting Information) were utilized to sample the designed continuous phase profiles. The selected unit cells demonstrate sufficient sampling capabilities, as depicted in Figure S5g. Due to the large area of our designed device, only a small portion of the sampling results are displayed.

7. Intuitive illustration of the operating principle of the cascaded metasurfaces.

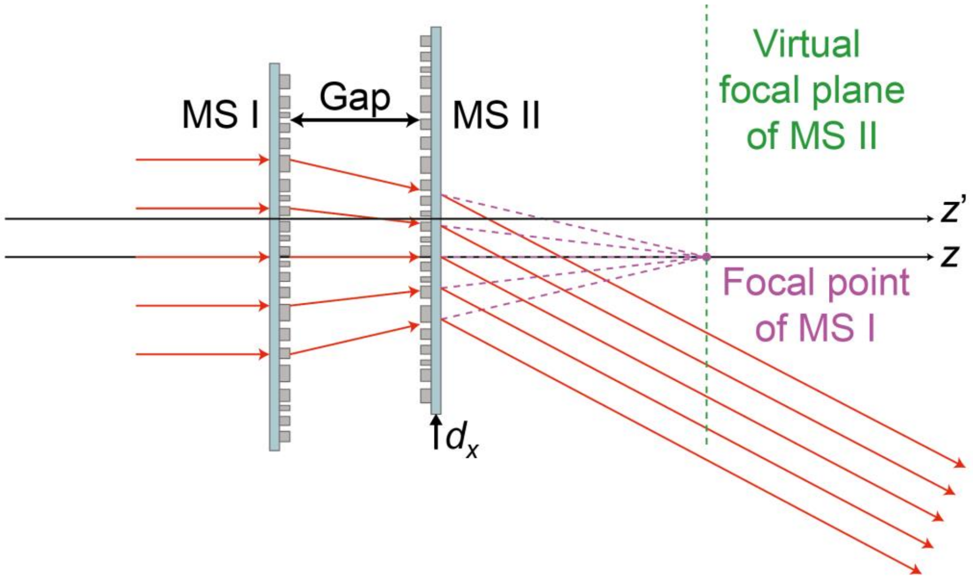

Figure S6 | Intuitive illustration of the operating principle of the cascaded metasurfaces when $p = q$ and the paraxial approximation is satisfied.

Under the paraxial approximation, the phase profile of a thin lens with a focal length f can be expressed as $\varphi_f = -\frac{k_0}{2f}(x^2 + y^2) = -\frac{\pi}{\lambda_0 f}(x^2 + y^2)$. The input metasurface MS I, with either a phase profile of φ_0 (f_0 = 6.98 mm) or φ_1 (f_1 = 4.92 mm), acts as a convex lens that focuses the incident wave to its focal point. The output metasurface MS II with φ_2 (f_2 = -4.92 mm) functions as a concave lens that collimates and steers the wave emanating from the virtual point source created by MS I. If the focal point of MS I coincides exactly with the virtual focal plane of MS II, as shown in Figure S6, the output wave from MS II will exhibit minimum divergence, ensuring optimal performance of the cascaded metasurface device. Otherwise, the collimation capability of the device will be degraded.

For cascaded φ_1 and φ_2, as $|f_1| = |f_2|$, the device operates optimally only when the gap size is close to 0, and an oversized gap will cause unwanted divergence. On the other hand, for cascaded φ_0 and φ_2, since $|f_0| > |f_2|$, the device can operate optimally at a gap size that is easily achievable. The optimal gap size can be calculated as $|f_0| - |f_2|$, which is only decided by the coefficients of their respective quadratic phase profiles and is independent of the metasurface dimensions.

However, the aforementioned analysis is only applicable under limited conditions where $p = q$ and the paraxial approximation is satisfied, whereas the proposed design principle of phase addition and reverse ray-tracing can be applied to a much broader range of cases.

8. Analysis of the maximum size of the metasurface devices.

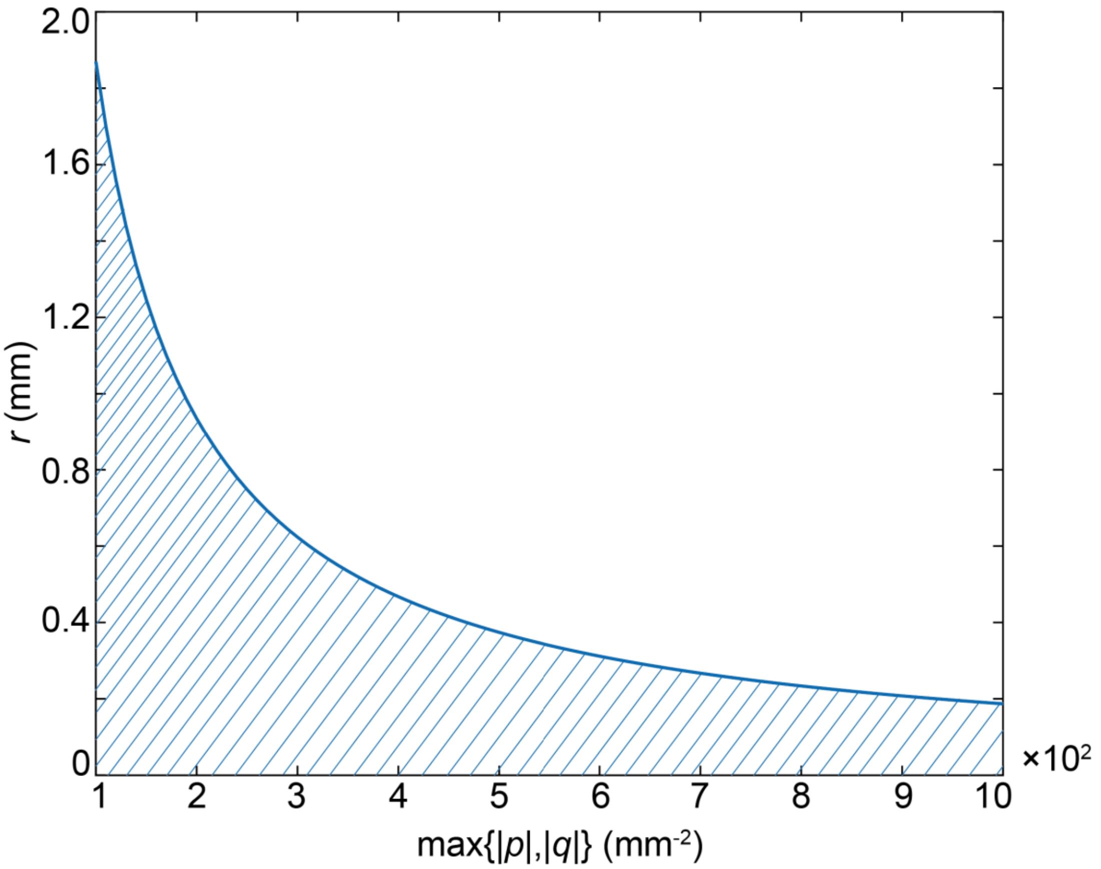

Figure S7 | Feasible range of scale factors (*p* and *q*) in front of the quadratic term of metasurface phase profiles, and the metasurface diameter (*r*). The blue shaded area corresponds to the feasible design range of *p*, *q* and *r* when the lattice constant *P* = 560 nm and the number of unit cells used to sample a phase variation of 2π is greater than M_{min} = 3.

For $\varphi_1(x,y) = -\varphi_2(x,y) = px^2 + qy^2$, the magnitude of the phase gradient is:

$$|Grad| = \sqrt{(2px)^2 + (2qy)^2} = 2\sqrt{p^2x^2 + q^2y^2} \qquad \text{(S24)}$$

For a metasurface with a circular shape of radius *r*, the maximum phase gradient magnitude appears at the edge of the device ($x^2 + y^2 = r^2$):

$$\begin{aligned} |Grad|_{x^2+y^2=r^2} &= 2\sqrt{p^2x^2 + q^2y^2} \\ &= 2\sqrt{p^2x^2 + q^2(r^2 - x^2)} \end{aligned} \qquad \text{(S25)}$$

Since *p* and *q* can be arbitrarily real numbers chosen by the designer to realize desired tunable responses, it is uncertain which of them is larger and which is smaller.

If $|p| \geq |q|$, then let $x^2 = r^2$, thus obtaining $|Grad|_{max} = 2|p|r$.

If $|p| < |q|$, then let $x^2 = 0$, thus obtaining $|Grad|_{max} = 2|q|r$.

Hence:

$$|Grad|_{max} = 2r \times max\{|p|, |q|\} \qquad \text{(S26)}$$

The minimum number of unit cells used to sample a phase variation of 2π at the metasurface edge can be estimated as:

$$M = \frac{2\pi}{2Pr \times max\{|p|, |q|\}} = \frac{\pi}{Pr \times max\{|p|, |q|\}} \qquad \text{(S27)}$$

where P is the lattice constant of the unit cell.

If we set $M > M_{min}$, then:

$$r < r_{max} = \frac{\pi}{PM_{min} \times max\{|p|, |q|\}} \qquad \text{(S28)}$$

For P = 560 nm and M_{min} = 3, the feasible range of r is plotted as the shaded area in Figure S7. For example, if $|p| = |q|$ = 423 mm^{-2}, then r should not exceed 4.42 mm; if $|p| = |q|$ = 600 mm^{-2}, then r should not exceed 3.12 mm.

For the experimental implementation of cascaded metasurfaces, MS I remains stationary relative to the light source and needs to cover only the incident beam spot, while MS II should be as large as possible to achieve wider tuning ranges of the direction angles. Thus, we choose diameter sizes of 4 mm for MS I (φ_0 and φ_1) and 6 mm for MS II (φ_2).

9. Analysis of the attainable tunable ranges of α and β.

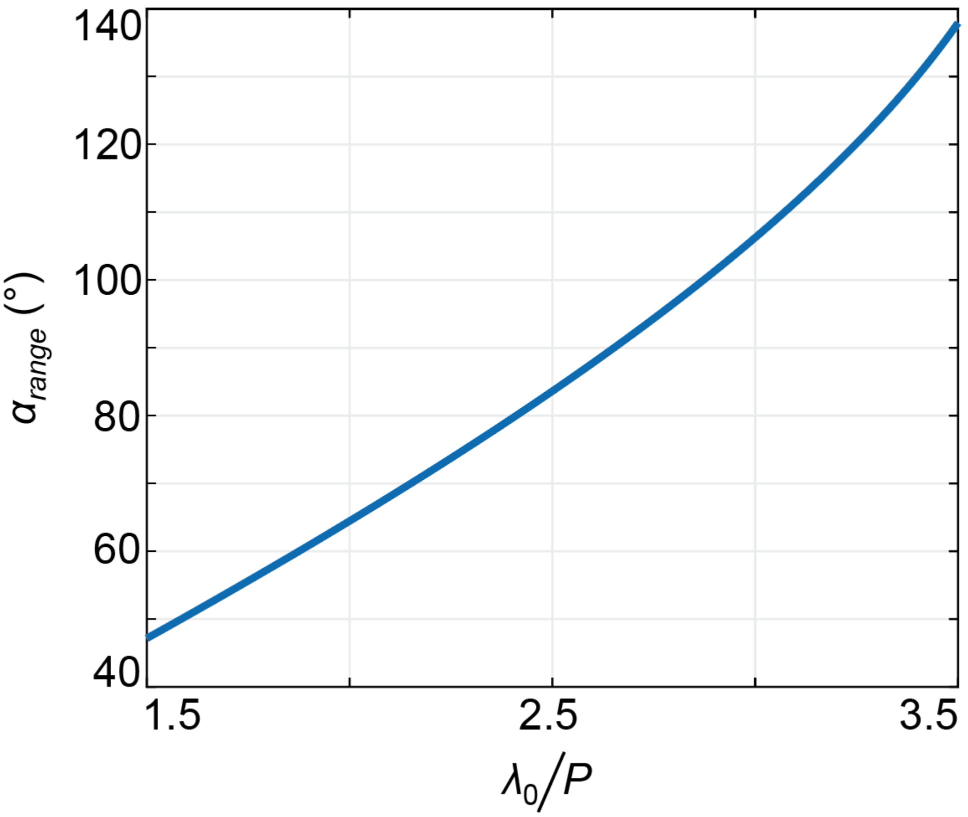

Figure S8 | The relationship between the theoretically attainable tunable range α_{range} and the metasurface spatial phase modulation fineness λ_0 / P when the translation ratio u = 0.8 and M_{min} = 3.

According to equations (4) and (5), the tunable ranges of α and β can be calculated as:

$$\alpha_{range} = \left| arccos\left(\frac{p\lambda_0}{\pi} d_{xmax}\right) - arccos\left(\frac{p\lambda_0}{\pi} d_{xmin}\right) \right| \quad \text{(S29)}$$

$$\beta_{range} = \left| arccos\left(\frac{q\lambda_0}{\pi} d_{ymax}\right) - arccos\left(\frac{q\lambda_0}{\pi} d_{ymin}\right) \right| \quad \text{(S30)}$$

where d_{xmin}, d_{xmax}, d_{ymin} and d_{ymax} represent the translation ranges of the output metasurface (MS II), i.e., $d_{xmin} \leq d_x \leq d_{xmax}$ and $d_{ymin} \leq d_y \leq d_{ymax}$. Given the axial symmetry of the designed quadratic phase profiles around the x- and y-axis, as well as the limited in-plane translation of MS II due to its maximum aperture size (refer to Section 8 of the Supporting Information), it is reasonable to assume:

$$\begin{aligned} d_{xmax} &= -d_{xmin} = ur_{max} \\ d_{ymax} &= -d_{ymin} = vr_{max} \end{aligned} \quad \text{(S31)}$$

where u, v are two constant numbers determining the ratio of the translation ranges to the maximum device aperture. By substituting (S28) and (S31) into (S29) and (S30), we can derive the following equations:

$$\alpha_{range} = 2\left(90° - arccos\left(\frac{|p|\lambda_0}{\pi}d_{xmax}\right)\right) = 2\left(90° - arccos\left(\frac{\lambda_0 u|p|}{PM_{min}max\{|p|,|q|\}}\right)\right) \quad \text{(S32)}$$

$$\beta_{range} = 2\left(90° - arccos\left(\frac{|q|\lambda_0}{\pi}d_{ymax}\right)\right) = 2\left(90° - arccos\left(\frac{\lambda_0 v|q|}{PM_{min}max\{|p|,|q|\}}\right)\right) \quad \text{(S33)}$$

For simplified situation where $p = q$ and $u = v$,

$$\alpha_{range} = \beta_{range} = 2\left(90° - arccos\left(\frac{\lambda_0 u}{PM_{min}}\right)\right) \quad \text{(S34)}$$

It is observed that the beam steering ranges are decided once the values of the four parameters λ_0, u, P, and M_{min} are settled. In our proposed design, we choose λ_0 = 1064 nm, u = 3.5/3.12, P = 560 nm and M_{min} = 3, resulting in $\alpha_{range} = \beta_{range}$ = 90.55°. A larger value of u or a smaller value of P or M_{min} will lead to an increase in the tunable ranges of α and β.

When applying the principle of the cascaded metasurfaces to other frequency bands in general, we may conclude that the spatial phase modulation fineness, i.e., λ_0 / P, plays a crucial role in determining the tunable steering ranges, as shown in Figure S8.

10. Forward ray-tracing simulations of the actual devices and measured light source properties.

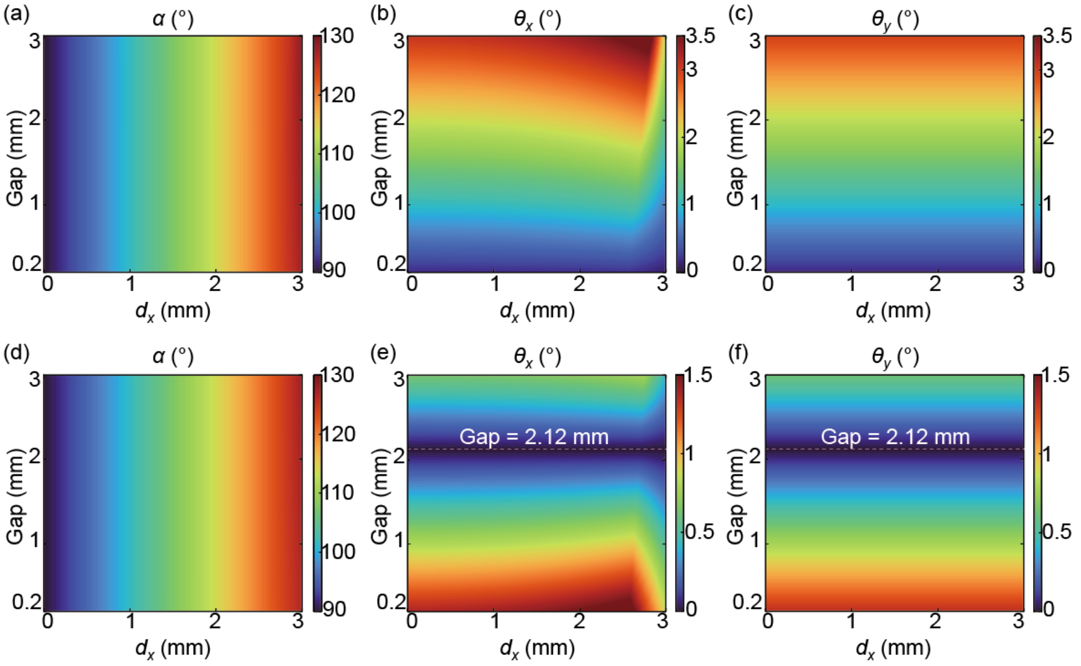

Figure S9 | Forward ray-tracing simulation results of the actual cascaded metasurfaces under the conditions of the measured light incidence. (a, b and c) Direction angle α and divergence angles θ_x and θ_y when MS I and MS II have phase profiles φ_1 and φ_2. (d, e and f) Direction angle α and divergence angles θ_x and θ_y when MS I and MS II have phase profiles φ_0 and φ_2.

The FWHM profile of the light spot hitting MS I was measured to be an ellipse with a half long axis length of 440 μm along the x direction and a half short axis length of 425 μm along the y direction, as shown in Figure 5g (left) and illustrated in the Methods section. Measured x and y divergence angles are 0.0310° and 0.0308°, respectively. According to these results, we perform another round of forward ray-tracing simulations in which we set a group of light rays emitted from a point source located 0.8 m away from MS I that reach MS I as an ellipse of the same size as our experimental measurements. We also consider the limited size of the real metasurfaces, such that any light rays that do not hit the effective region of the device continue to travel along the original direction and are excluded when calculating α, θ_x and θ_y. Thus, simulation results in Figure S9 incorporate considerations of real experimental conditions compared to Figure 3. The gap size for minimal divergence of deflected light increases from 2.05 mm to 2.12 mm due to the slight divergence of incident beam, and a reduced effective aperture size causes θ_x to decrease at large d_x. The results are also demonstrated in Movie S3.

11. Effect of a reduced effective aperture size.

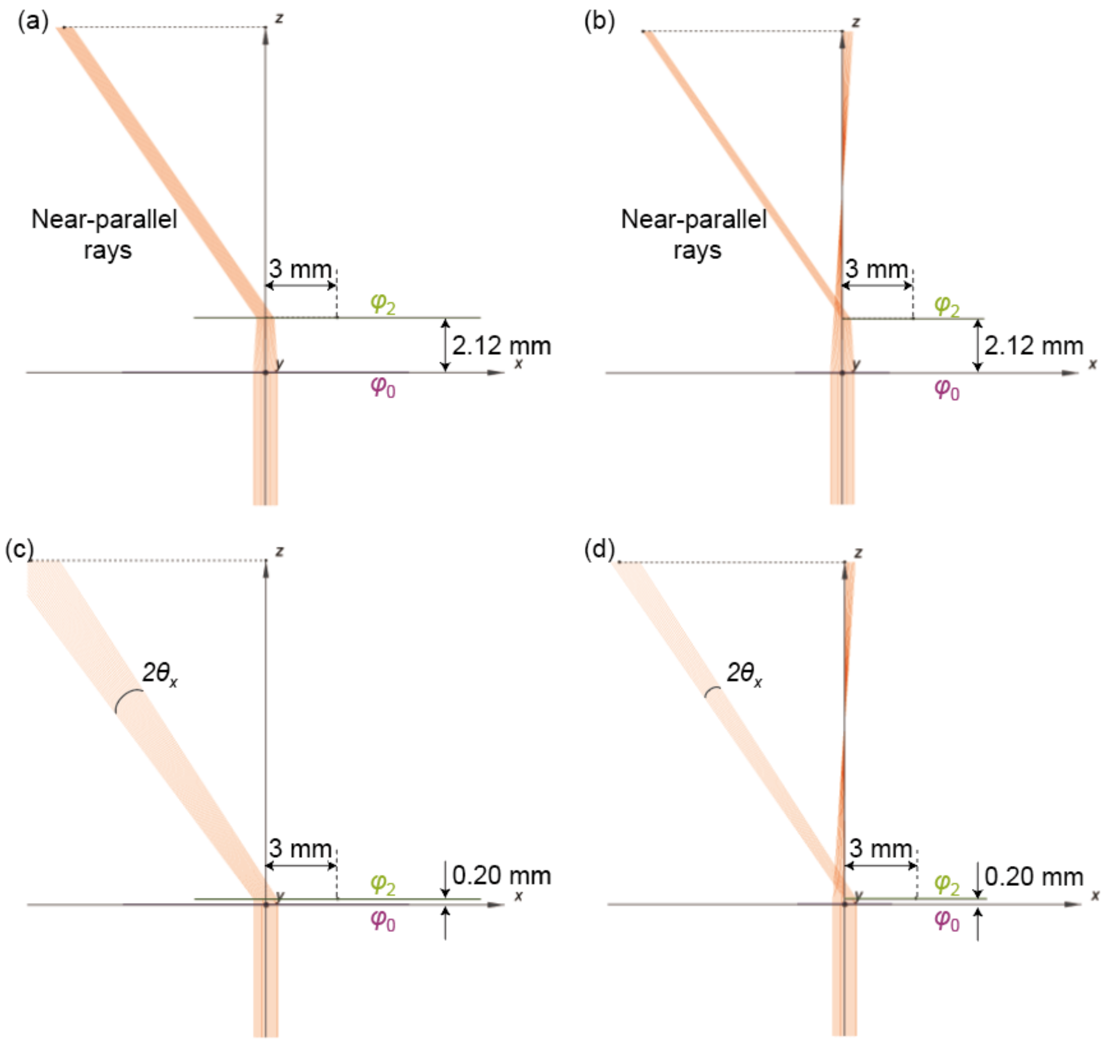

Figure S10 | Effect of a reduced effective aperture size. Side views of the forward ray-tracing simulations of the cascaded metasurfaces with large diameters (a and c) and real diameters (b and d), and separated by the optimal gap of 2.12 mm (a and b) and a randomly chosen gap of 0.20 mm (c and d) at a large translation d_x of 3 mm. The metasurfaces have transmission phase profiles φ_0 and φ_2, and the deflected beam has a x divergence angle θ_x.

The effect of a reduction in effective aperture size is simulated and compared, as shown in Figure S10. If the cascaded metasurfaces operate at the optimal gap size (Figure S10a and S10b), the reduction in effective aperture size causes the reduction of deflected beam size, but the deflected beam still exhibits near zero divergence. If the cascaded metasurfaces operate at a suboptimal gap size (Figure S10c and S10d), the reduction in effective aperture size causes the reduction of deflected beam size, and the divergence angle values of the deflected beam decrease accordingly. We may conclude that the closer the gap size is to its optimal value, the smaller the effect of the reduction in effective aperture size on the divergence angles will be.

However, above analysis is based on the geometric optics, while diffraction and scattering cannot be neglected when part of light reaching MS II plane lies outside the effective metasurface region, which introduces unwanted stray light and extra beam divergence. We may assume that the effect of diffraction and scattering increases as d_x increases with a fixed gap size, but remains similar at various gap sizes (1.5mm – 2.5 mm) with a fixed d_x.

This provides us with new perspectives to explain the measurement results of θ_x (Figure 5e). At the optimal gap sizes of 2.12 mm, as d_x increases and the effective aperture size decreases, the reduction of deflected beam size doesn't result in the reduction of θ_x, but the diffraction and scattering induce larger and larger θ_x. At the gap sizes close to the optimal value (1.90 mm and 2.30 mm), as d_x increases and the effective aperture size decreases, the reduction of deflected beam size leads to the reduction of θ_x, but the effect is always smaller than that of diffraction and scattering. Thus, θ_x monotonically increases as d_x increases. At gap values far from the optimal value (1.50 mm, 1.70 mm and 2.50 mm), as d_x increases and the effective aperture size decreases, the reduction of deflected beam size leads to the reduction of θ_x and overweighs the effect of diffraction and scattering when reaching a certain d_x. Thus, θ_x increases then decreases as d_x increases.

12. Optical efficiency measurement results of the demonstrated device.

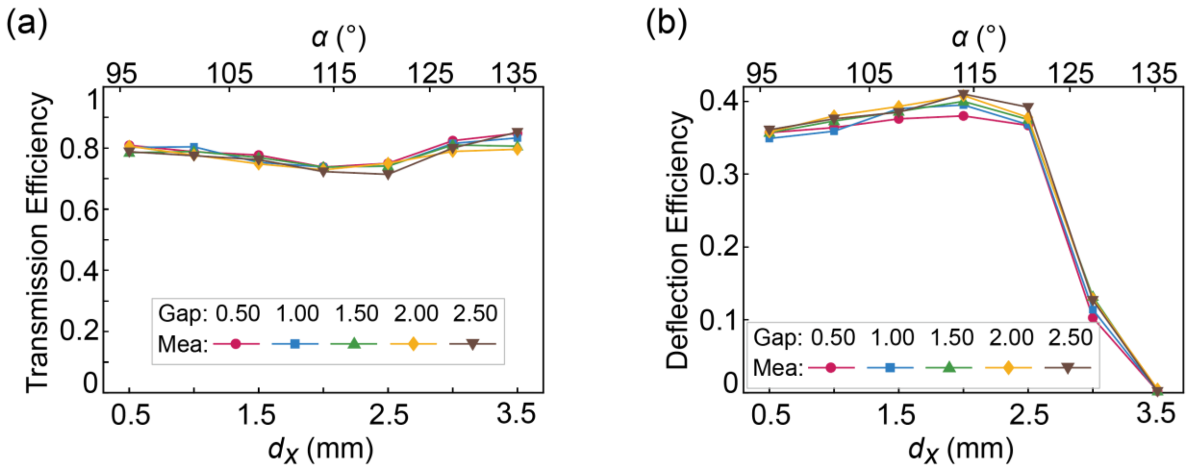

Figure S11 | Measurement results of transmission efficiency and deflection efficiency for cascaded φ_0 (MS I) and φ_2 (MS II) as functions of d_x (α) at multiple gap sizes (mm).

The transmission efficiency is defined as the ratio of transmitted beam power to incident beam power, and the deflection efficiency is defined as the ratio of deflected beam power to transmitted beam power. In this way, the overall optical efficiency is the product of the transmission efficiency and the deflection efficiency. The measured results of the three types of optical efficiency are plotted in Figure S11 and 5h.

The proposed device's optical efficiency may be limited by several factors, with the fabrication imperfections being the primary concern. Another factor is the refection that occurs at the interfaces between the substrate and the ambient air. Accordingly, we may implement a more meticulous fabrication process and deposit an antireflection coating on the substrate to enhance the optical efficiency.

13. Numerical simulation of the side mode suppression ratio (SMSR) of the cascaded metasurfaces.

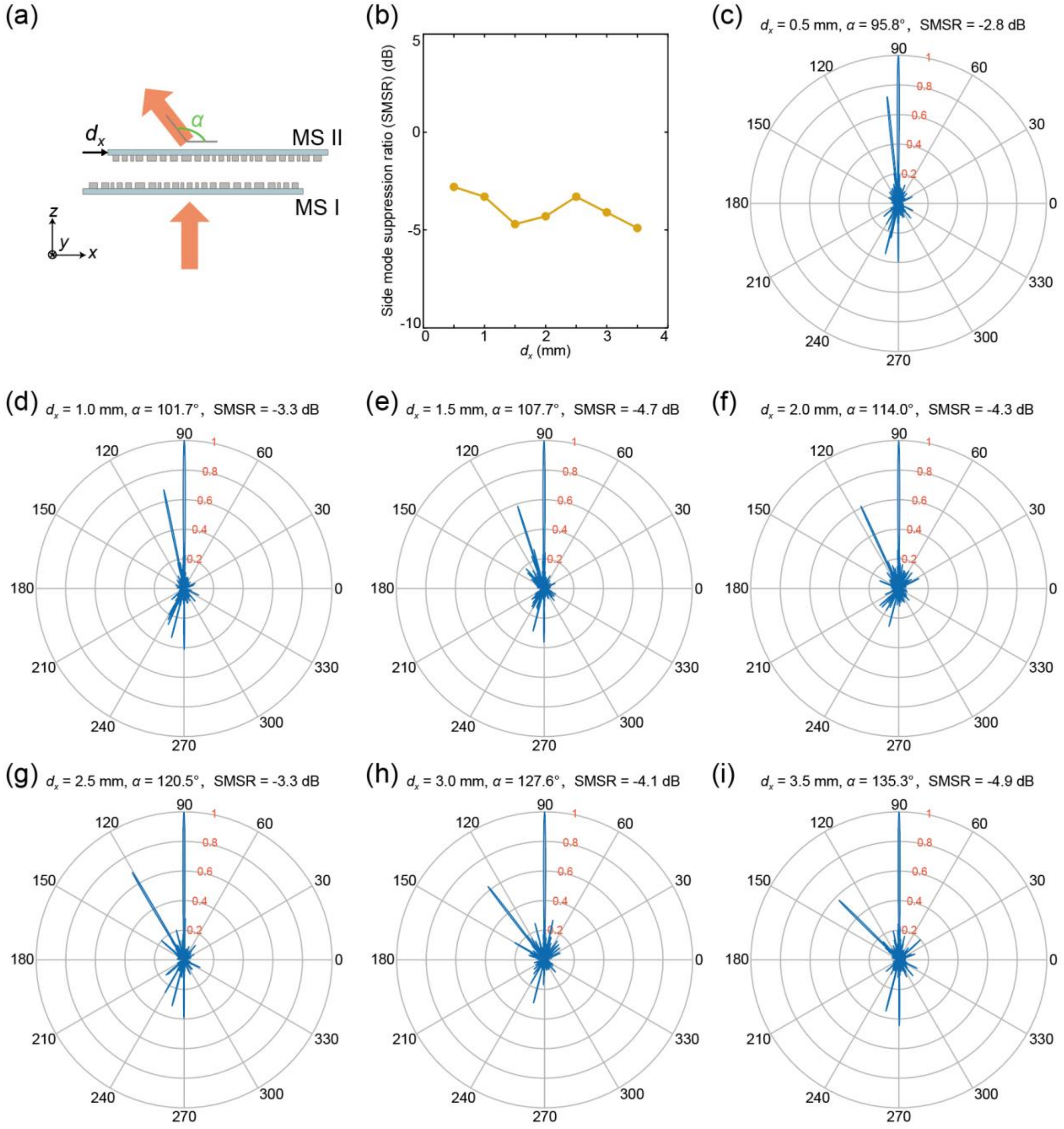

Figure S12 | Numerical simulation of the side mode suppression ratio (SMSR) of the cascaded metasurfaces. (a) Schematic of the simulation setup. (b) Simulated SMSR as a function of the in-plane displacement d_x. (c – i) Simulated normalized far-field E-field patterns for various d_x values.

Due to the symmetry of our metasurfaces, we only simulated the responses when α increased from 90° to 135°. As shown in Figure S12, the main lobe is the steered beam in the desired direction, and the highest side lob is the transmitted beam in the incoming direction.

The low simulated SMSR may be attributed to the large periodicity of the metasurface arrays, and the imperfections in the amplitude and phase responses of the unit cells. Limited by the fabrication capability, we chose the periodicity in our metasurfaces as 560 nm. In order to improve the SMSR and deflection efficiency, we may decrease the periodicity to improve the diffraction efficiency. Furthermore, the amplitude of each individual unit cell exhibits non-uniformity and the phase response may undergo slight alterations when arranged in a non-uniform array. Therefore, the SMSR may be further improved by systematic optimization of the overall metasurface array and finer tune of the amplitude and phase responses.

References:

[51] W. Suh, Z. Wang, S. H. Fan, *Ieee Journal of Quantum Electronics* **2004**, 40, 1511.

[52] S. H. Fan, W. Suh, J. D. Joannopoulos, *Journal of the Optical Society of America a-Optics Image Science and Vision* **2003**, 20, 569.
